# Relationship between institutional ventilated COVID-19 case volume and in-hospital death: A multicenter cohort study

**DOI:** 10.1371/journal.pone.0287310

**Published:** 2023-06-15

**Authors:** Shunsuke Amagasa, Satoko Uematsu, Mitsuru Kubota, Masahiro Kashiura, Hideto Yasuda, Mineji Hayakawa, Kazuma Yamakawa, Akira Endo, Takayuki Ogura, Atsushi Hirayama, Hideo Yasunaga, Takashi Tagami

**Affiliations:** 1 Division of Emergency and Transport Services, National center for Child Health and Development, Okura, Setagaya-ku, Tokyo, Japan; 2 Department of Interdisciplinary Medicine, Division of General Pediatrics, National Center for Child Health and Development, Okura, Setagaya-ku, Tokyo, Japan; 3 Department of Emergency and Critical Care Medicine, Saitama Medical Center, Jichi Medical University, Amanuma-cho, Omiya-ku, Saitama City, Saitama, Japan; 4 Department of Emergency Medicine, Hokkaido University Hospital, Kita-ku, Sapporo, Japan; 5 Department of Emergency Medicine, Osaka Medical and Pharmaceutical University, Daigakumachi, Takatsuki, Osaka, Japan; 6 Trauma and Acute Critical Care Center, Tokyo Medical and Dental University Hospital, Yushima, Bunkyo-ku, Tokyo, Japan; 7 Department of Emergency Medicine and Critical Care Medicine, Tochigi Prefectural Emergency and Critical Care Centre, Imperial Foundation Saiseikai Utsunomiya Hospital, Takebayashi-machi, Utsunomiya, Tochigi, Japan; 8 Department of Social Medicine, Public Health, Graduate School of Medicine, Osaka University, Yamadaoka, Suita, Japan; 9 Department of Clinical Epidemiology and Health Economics, School of Public Health, The University of Tokyo, Hongo, Bunkyo-ku, Tokyo, Japan; 10 Department of Emergency and Critical Care Medicine, Nippon Medical School Musashikosugi Hospital, Kosugimachi, Nakahara-ku, Kawasaki, Kanagawa, Japan; Azienda Ospedaliero Universitaria Careggi, ITALY

## Abstract

**Background:**

The volume-outcome relationship in patients with severe Coronavirus disease 2019 (COVID-19) is unclear and is important for establishing a system for the medical care of severe COVID-19. This study aimed to evaluate the association between institutional case volume and outcomes in patients with ventilated COVID-19.

**Methods:**

We analyzed patients with severe COVID-19 on ventilatory control aged > 17 years who were enrolled in the J-RECOVER study, which is a retrospective multicenter observational study conducted between January 2020 and September 2020 in Japan. Based on the ventilated COVID-19 case volume, the higher one-third of institutions were defined as high-volume centers, the middle one-third as middle-volume centers, and the lower one-third as low-volume centers. The primary outcome measure was in-hospital mortality during hospitalization due to COVID-19. Multivariate logistic regression analysis for in-hospital mortality and ventilated COVID-19 case volume was performed after adjusting for multiple propensity scores and in-hospital variables. To estimate the multiple propensity score, we fitted a multinomial logistic regression model, which fell into one of the three groups based on patient demographics and prehospital factors.

**Results:**

We analyzed 561 patients who required ventilator management. In total, 159, 210, and 192 patients were admitted to low-volume (36 institutions, < 11 severe COVID-19 cases per institution during the study period), middle-volume (14 institutions, 11–25 severe cases per institution), and high-volume (5 institutions, > 25 severe cases per institution) centers, respectively. After adjustment for multiple propensity scores and in-hospital variables, admission to middle- and high-volume centers was not significantly associated with in-hospital death compared with admission to low-volume centers (adjusted odds ratio, 0.77 [95% confidence interval (CI): 0.46–1.29] and adjusted odds ratio, 0.76 [95% CI: 0.44–1.33], respectively).

**Conclusions:**

There may be no significant relationship between institutional case volume and in-hospital mortality in patients with ventilated COVID-19.

## Introduction

Coronavirus disease 2019 (COVID-19) was first reported as an outbreak of pneumonia in Wuhan, a city in Hubei Province, China, at the end of 2019, and severe acute respiratory syndrome coronavirus 2 (SARS-CoV2) was confirmed to be the cause [1,2]. Subsequently, COVID-19 caused a global pandemic in March 2020 [3,4]. Since then, the pandemic has not abated, with more than 491 million people infected worldwide, and currently, more than 6.1 million deaths have been reported (as of April 4, 2022). COVID-19 causes many serious injuries, and 5%-20% of infected patients become severely ill and require treatment in intensive care units (ICU) [1,4]. Mortality rates for critically ill patients with COVID-19 are between 15% and 61% despite treatment in the ICU [5–8].

High-quality, specialized medical approaches are required for patients experiencing a critical illness or severe trauma and those requiring certain procedures, and volume-outcome relationships in these patients have been previously reported [9,10]. For various surgeries and conditions, including critical care, mechanical ventilation, pancreatic cancer, esophageal cancer, abdominal aortic aneurysm, and coronary artery bypass surgery, the larger the hospital case volume, the better the prognosis. Few studies have examined the volume-outcome relationship in critically ill patients with COVID-19, and the effect of case volume alone on outcomes in these cases remains unclear. In many of the regions that experienced the rapid expansion of COVID-19 earlier, the burden of disease was not uniform across regions due to restricted healthcare systems and limited resources [11]. In 2020, resources for intensive care treatment of severe COVID-19 were maintained in Japan [12]. A better understanding of the volume-outcome relationship in patients with severe COVID-19 in less healthcare-pressured areas could help create an efficient healthcare system and improve care and outcomes for patients with severe COVID-19.

To evaluate the volume-outcome relationship in patients with severe COVID-19 in areas where resources for critical care are maintained, we investigated the association between institutional case volume and outcome in ventilated critically ill patients with COVID-19.

## Materials and methods

### Study design and setting

The J-RECOVER study was a retrospective multicenter observational study conducted between January and September 2020 in Japan. The design, data collection methods, and protocols for the J-RECOVER study have been reported in detail previously [13]. This study was approved by the institutional ethics committee of each participating institution, with a waiver for informed consent to protect participant anonymity.

### Patients

All patients with COVID-19 admitted to the participating institutions during the study period were included in the J-RECOVER study. The current study included patients with severe COVID-19 on ventilatory control aged >17 years who were enrolled in the J-RECOVER study.

### Data collection

The existing clinical information of patients was obtained from diagnosis procedure combination (DPC) data and medical records. The DPC system is a comprehensive evaluation system for medical fees for acute inpatient care. Data were claimed to have been created and stored electronically at each facility under a comprehensive payment system based on DPC [13,14]. DPC data included sex, birth date, the main purpose of care during hospitalization, admission date, discharge date, patient transfer, route of admission, hospital referral or outpatient department admission, scheduled or emergency care admission, ambulance transport, discharge destination, and discharge outcome. Other necessary information that could not be obtained from the DPC data was obtained from the medical records of the institution and its personnel.

### Measurements and definitions

The primary outcome measurement was in-hospital death during hospitalization with COVID-19.

Hospital volume was defined as the number of eligible patients treated at each hospital, and it was subcategorized into tertials, as per previous studies [15–17]. The cutoff value was defined to determine the number of patients as equally as possible. The higher one-third of institutions were defined as high-volume centers, the middle one-third as middle-volume centers, and the lower one-third as low-volume centers. The Charlson comorbidity index was used as a measure of underlying disease [18]. The Sequential Organ Failure Assessment (SOFA) score was used as the severity score [19].

### Statistical analysis

We compared demographic factors, patient characteristics, hospital care, and outcomes among patients treated in high-, middle-, and low-volume centers. Trends in discrete variables among the three groups were analyzed using the Mantel–Haenszel trend test, and continuous variables were analyzed using the Kruskal–Wallis test.

Missing data were accounted for by undertaking multiple imputations to minimize bias and maximize the power of analyses [20]. Twenty multiple imputed datasets were generated, and the association between the severe COVID-19 case volume and in-hospital death in each dataset was evaluated using multivariate logistic regression analysis. The estimates were then combined.

We used multiple propensity score analysis in the multivariate analysis to adjust for confounding factors before admission that could be measured [21,22]. A propensity score represents the conditional probability of a particular exposure given a set of measured baseline covariates and is an established method for reducing confounding factors in observational studies. Although propensity score matching or stratification has commonly been used to assemble similar patient cohorts in terms of baseline covariates for 2-group comparisons, a multiple propensity score approach has been used for comparisons of three or more groups [22–24]. A multiple propensity score is defined as the conditional probability of being categorized into a particular group of three or more given a set of observed baseline covariates. To estimate the multiple propensity score for the current analysis, we fitted a multinomial logistic regression model, categorized into one of the three groups along with hospital characteristics, including teaching status, number of hospital beds, number of ICU beds, number of annual ICU admissions (2019), patient demographics (age, sex, and underlying disease), and prehospital factors (number of days from symptom onset to hospitalization, number of days from positive polymerase chain reaction test to hospitalization, transfer from another hospital, and severity of illness on admission). Next, we used multivariate logistic regression to analyze the association between institutional severe COVID-19 case volume and in-hospital mortality with adjustment for multiple propensity scores and in-hospital variables, including favipiravir, remdesivir, ICU admission, use of prone position, and use of extracorporeal membrane oxygenation (ECMO).

We also performed a sensitivity analysis using data from cases where all necessary values were recorded for robustness. This sensitivity analysis was performed with multivariate logistic regression analysis of institutional volume and in-hospital mortality adjusted for multiple propensity scores calculated from preadmission confounders and in-hospital variables as in the main analysis.

All statistical tests were two-sided, and a level of 0.05 was considered statistically significant. All data analyses were performed using SPSS statistical software package version 24 (IBM, New York, USA).

## Results

### Patients

**Fig 1:**
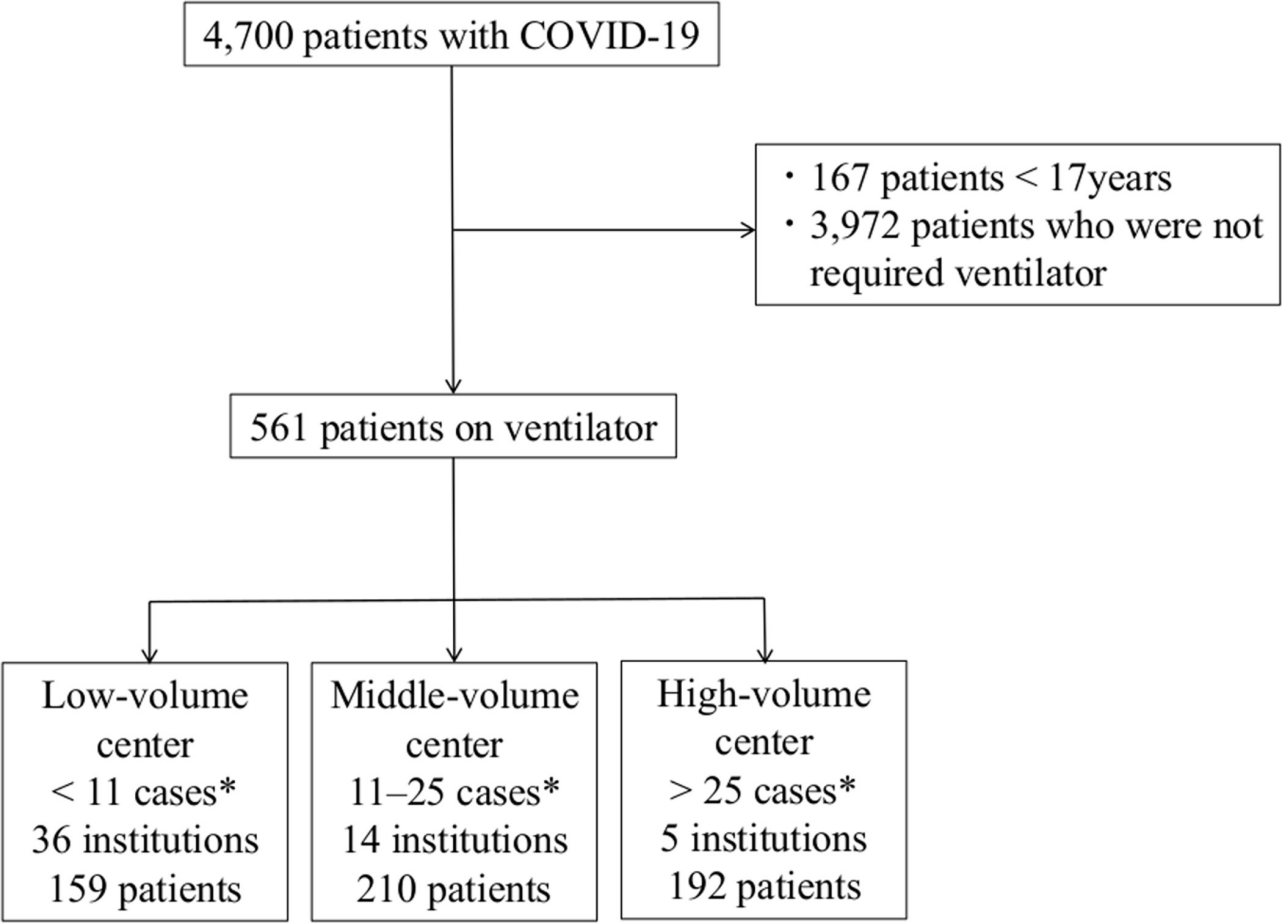
Flowchart of the study. *During the study period (9 months).

In total, 4,700 patients with COVID-19 from 66 institutions were enrolled in the J-RECOVER study. Among these patients, 561 were adults with COVID-19 who required ventilator management (Fig 1). None of the patients met the exclusion criteria. A total of 159, 210, and 192 patients were admitted to low-volume (36 institutions, < 11 severe COVID-19 cases per institution during the study period), middle-volume (14 institutions, 11–25 severe cases per institution), and high-volume (5 institutions, >25 severe cases per institution) centers, respectively. There were no ventilated patients in 11 institutions.

### Institutional information and patient characteristics

There were no significant differences in the number of academic hospitals, hospital beds, ICU beds, or ICU admissions in 2019 among the three groups (Tables 1 and 2). The number of ECMO patients per institution in low-volume centers was significantly lower than that in the other two groups.

**Table 1:** Institutional information.

	Low-volume center (n = 159)	Middle-volume center (n = 210)	High-volume center (n = 192)	*p*
Number of institutions	36	14	5	
Number of academic hospitals, n (%)	21 (58.3)	6 (42.9)	2 (40.0)	0.28
Number of hospital beds, median (IQR)	689 (495–894)	701 (634–908)	799 (749–1009)	0.88
Number of ICU beds, median (IQR)	27 (17–42)	30 (15–40)	34 (11–50)	0.82
Number of ICU admissions in 2019, median (IQR)†	1750 (888–2600)	2122 (918–2355)	2059 (689–3828)	0.91
Number of patients per institution, median (IQR)	4 (2–6)	18 (12–20)	40 (27–53)	< 0.001
Number of ECMO patients per institution, median (IQR)	1 (0–1.8)	2.5 (0.8–6)	3 (1–5)	0.01

IQR, interquartile range; ICU, intensive care unit.

†ICU admissions from January to December 2019.

**Table 2:** Characteristics of the study population.

	Missing data	Low-volume center (n = 159)	Middle-volume center (n = 210)	High-volume center (n = 192)	*p*
Age, median (IQR)	0	66 (57–75)	63 (53–70)	71 (57–77)	< 0.001
Sex, male, n (%)	0	30 (18.9)	40 (19.0)	49 (25.5)	0.20
Transfer from another hospital, n (%)	0	75 (47.2)	97 (46.2)	136 (70.8)	< 0.001
Charlson Comorbidity index	0				0.13
0, n (%)		98 (61.6)	126 (60.0)	131 (68.2)	
1, n (%)		35 (22.0)	58 (27.6)	43 (22.4)	
2, n (%)		16 (10.1)	17 (8.1)	11 (5.7)	
3, n (%)		6 (3.8)	6 (2.9)	2 (1.0)	
4 or more, n (%)		4 (2.5)	3 (1.4)	5 (2.6)	
Number of days from symptom onset to hospitalization, median (IQR)	13	7 (4–10)	8 (5–11)	8 (5–10)	0.11
Number of days from PCR test to hospitalization, median (IQR)	100	1 (0–5)	2 (0–6)	2 (0–5)	0.35
SOFA score on admission, median (IQR)	124	3 (2–6)	5 (3–8)	4 (3–6)	0.17
Favipiravir administration, n (%)	0	114 (71.7)	142 (67.6)	156 (81.3)	0.03
Remdesivir administration, n (%)	0	41 (25.8)	53 (25.2)	43 (22.4)	0.45
ICU admission, n (%)	0	148 (93.1)	201 (95.7)	189 (98.4)	0.01
Prone position, n (%)	37	38 (26.6)	86 (42.6)	79 (44.1)	0.002
ECMO, n (%)	0	36 (22.6)	51 (24.3)	15 (7.8)	< 0.001

IQR, interquartile range; PCR, positive polymerase chain; SOFA, Sequential Organ Failure Assessment; ICU, intensive care unit; ECMO, extracorporeal membrane oxygenation.

The age of the patients in the high-volume center was significantly higher than in the other two groups (< 0.001). Patients in high-volume centers had a higher probability of transfer from another hospital (p < 0.001), favipiravir administration (p = 0.031), ICU admission (p = 0.01), and a lower probability of ECMO (p < 0.001). Patients in low-volume centers had a lower probability of a prone position (p = 0.002).

### The difference in outcomes among institutions

The length of stay in the hospital and ICU was significantly shorter as the case volume increased. In-hospital death was not significantly different among the three groups (Table 3).

**Table 3:** The difference in outcomes among institutions.

	Missing data	Low-volume center (n = 159)	Middle-volume center (n = 210)	High-volume center (n = 192)	*p*
Transfer to another department, n (%)	0	53 (33.3)	66 (31.4)	65 (33.9)	0.25
Transfer to another hospital, n (%)	2	65 (40.9)	76 (36.2)	94 (49.0)	0.10
Length of stay in hospital, day, median (IQR)	0	25 (14–40)	20 (12.5–32)	18 (11–29)	0.001
Length of stay in ICU, day, median (IQR)	37	17 (9–28)	12 (7–18)	10 (7–18)	< 0.001
In-hospital death, n (%)	0	46 (28.9)	47 (22.4)	53 (27.6)	0.85

IQR, interquartile range.

### Multivariate logistic regression analyses for in-hospital death adjusted by multiple propensity score and in-hospital variables

After adjustment for multiple propensity scores and in-hospital variables, admission to middle- and high-volume centers was not significantly associated with in-hospital mortality compared with admission to low-volume centers (adjusted odds ratio, 0.77 [95% confidence interval (CI): 0.46–1.29] and adjusted odds ratio, 0.76 [95% CI: 0.44–1.33], respectively) (Table 4).

**Table 4:** Multivariate logistic regression analyses for in-hospital death adjusted by multiple propensity score and in-hospital variables.

	Crude OR (95% CI)	*p*	Adjusted OR (95% CI)	*p*
Institution				
Low-volume center	Reference		Reference	
Middle-volume center	0.71 (0.44–1.14)	0.15	0.77 (0.46–1.29)	0.32
High-volume center	0.94 (0.59–1.49)	0.78	0.76 (0.44–1.33)	0.33

A multiple propensity score was defined as the conditional probability of falling into a particular group of three groups of institutional case volume. We fitted a multinomial logistic regression model, which fell into one of the three groups as patient demographics and prehospital factors.

### Sensitivity analysis of multivariate logistic regression analyses for in-hospital death adjusted by multiple propensity score and in-hospital variables using data of the cases with complete data

In the sensitivity analysis, admission to middle- and high-volume centers was also not significantly associated with in-hospital mortality compared with admission to low-volume centers (adjusted odds ratio, 0.76 [95% CI: 0.33–1.72] and adjusted odds ratio, 0.61 [95% CI: 0.29–1.30], respectively) (Table 5).

**Table 5:** Sensitivity analysis of multivariate logistic regression analyses for in-hospital death adjusted by multiple propensity score and in-hospital variables using data of the cases with complete data (n = 279).

	Adjusted OR (95% CI)	*p*
Institution		
Low-volume center	Reference	
Middle-volume center	0.76 (0.33–1.72)	0.50
High-volume center	0.61 (0.29–1.30)	0.20

A multiple propensity score was defined as the conditional probability of falling into a particular group of three groups of institutional case volume. We fitted a multinomial logistic regression model, which fell into one of the three groups as patient demographics and prehospital factors.

## Discussion

The present study evaluated the volume-outcome relationship in critically ill patients with ventilated COVID-19. In Japan, we did not observe a significant association between institutional case volume and in-hospital mortality after adjusting for in-hospital confounding factors and multiple propensity scores (calculated based on patient characteristics and prehospital confounding factors). The findings from this study may help in the construction of a medical system for patients with severe COVID-19.

There are many observational studies on volume-outcome relationships in critical care, and the relationship remains a matter of debate [16,17,25]. One systematic review of the volume-outcome relationship in critical care reported that high volumes might improve survival outcomes, although there is no complete consistency in this relationship [10]. Regarding the volume of mechanical ventilation, several studies showed that high volume improved outcomes, while others found no such association. Two studies with data before 2003 [26,27], one with an instrumental variable analysis conducted between 2004 and 2006 [28], and a more recent study conducted with patients with acute respiratory distress syndrome [15] reported that a higher case volume of mechanical ventilation improved outcomes. No association was found between volume outcomes in ventilated patients in two studies using data from 2008 and 2009 [29,30]. In contrast, a recent large retrospective cohort study showed that patient outcomes were worse at hospitals with higher mechanical ventilation volumes [31]. These differences in results may be due to the influence of changes in mechanical ventilatory management over time, differences in health care systems, types of hospitals, statistical methods, or adjusting for confounding factors. Furthermore, unlike previous studies that examined the volume outcome of other specific diseases, procedures, or surgeries, previous studies on critical care had a heterogeneous population with a wide range of patient diseases and treatments. This heterogeneity may have greatly influenced the discrepancy in the findings of the previous studies.

In the present study, we observed that the institutional ventilated COVID-19 case volume was not associated with in-hospital mortality. This study is a relatively small number of patients and institutions for a study examining volume-outcome relationships. Issues such as low variability in the number of patients among participating institutions and the small number of institutions included in the high-volume centers may be related to the results. However, our study examined a single disease, making our study less heterogeneous than previous studies of volume outcomes in critical care. Moreover, multiple imputations to address missing data and multiple propensity score analysis was undertaken, allowing adjustment for many confounding factors, including hospital and patient characteristics, prehospital factors, and treatment. Age, which is an important factor with respect to mortality and is skewed by group, was also adjusted for. We did not observe a volume-outcome relationship after multiple propensity score analysis, adjusting for these factors. To the best of our knowledge, there are no publications on the volume-outcome relationship for COVID-19, and we believe this study is important for examining the same.

There are several possible reasons for the lack of a significant association between the institutional volume of ventilated COVID-19 cases and outcomes. In the present study, many participating hospitals were tertiary emergency centers, and both high- and low-volume centers were habitually proficient in ventilator management and could provide higher-level care, such as ECMO, if necessary. In other words, there may be equalization of care with respect to the participating institutions in this study. It is also possible that even high-volume centers may not be able to improve outcomes because COVID-19 is an emerging infection, and care has not yet been established. However, the mortality rate was lower than that in other countries, indicating a superior quality of care. In the future, it will be necessary to observe whether the development of COVID-19 treatment affects the volume outcome. The higher-volume facilities might not have enough resources due to the sudden COVID-19; pandemic. However, no shortage of resources for intensive care in Japan during the pandemic was observed. Our data suggest that institutional ventilated COVID-19 case volume was not associated with survival outcomes in an ICU with adequate resources.

The group with a higher institutional case volume had significantly shorter ICU stays and hospitalizations. It was thought that a large number of transfers to other departments and discharges due to transfers to other hospitals might be associated with shorter ICU and hospital stays. However, transfers to another department did not differ among the three groups, and discharges due to transfer to other hospitals tended to be higher in high-volume centers; nevertheless, this was not significant. Shorter ICU and hospital stays may also be related to practice. The high-volume center could treat more patients with severe COVID-19 due to shorter ICU and hospital stays, which is important in terms of the effective utilization of resources.

This study had several limitations. First, the J-RECOVER study was conducted only at certain hospitals and was not a population-based study; therefore, the generalizability of the results may be limited. Second, although understanding volume-outcome relationships requires sufficient hospital numbers to create stable comparisons, the number of eligible institutions and cases was relatively low. The lack of a large difference in the number of patients between high-volume and low-volume institutions and the number of only five high-volume institutions may be related to the result that the volume-outcome relationship was not found. Because this study targeted a single disease and was not highly heterogeneous, the results are considered meaningful; however, further large-scale studies are needed. Third, this study included patients in the early stages of the COVID-19 pandemic as an emerging infectious disease. The results may change in the future as effective treatment and care becomes more established. The volume-outcome relationship of COVID-19 as an emerging infectious disease needs to be examined over time, considering changes in treatment methods and protocols. Fourth, this is a retrospective study, and caution should be exercised in its interpretation because of missing values, unmeasured confounding, and other issues. Future prospective studies on the volume-outcome relationship of emerging infectious diseases are needed.

## Conclusions

In the present study, institutional ventilated COVID-19 case volume was not associated with in-hospital death after adjusting for patient demographics and pre-hospital and in-hospital factors. The results of this study may aid in the construction of a medical system for patients with severe COVID-19. However, further investigation is needed to determine the association between institutional case volumes and outcomes in patients with severe COVID-19.
